# The Genotypic and Phenotypic Stability of *Plasmodium falciparum* Field Isolates in Continuous *In Vitro* Culture

**DOI:** 10.1371/journal.pone.0143565

**Published:** 2016-01-11

**Authors:** Redemptah Yeda, Luicer A. Ingasia, Agnes C. Cheruiyot, Charles Okudo, Lorna J. Chebon, Jelagat Cheruiyot, Hoseah M. Akala, Edwin Kamau

**Affiliations:** 1 Department of Emerging Infectious Diseases-Global Emerging Infections Surveillance and Response System (DEID-GEIS) Program, United States Army Medical Research Directorate-Kenya (USAMRD-K), Kenya Medical Research Institute (KEMRI)-Walter Reed Project, Kisumu, Kenya; 2 Institute of Tropical Medicine and Infectious Diseases, College of Health Sciences, Jomo Kenyatta University of Agriculture and Technology, Nairobi, Kenya; Centers for Disease Control and Prevention, UNITED STATES

## Abstract

The *Plasmodium falciparum in vitro* culture system is critical for genotypic and phenotypic analyses of the parasites. For genotypic analysis, the genomic DNA can be obtained directly from the patient blood sample or from culture adapted parasites whereas for phenotypic analysis, immediate *ex vivo* or *in vitro* culture adapted parasites are used. However, parasite biology studies have not investigated whether culture adaptation process affects genotypic and/or phenotypic characteristics of the parasites in short- or long-term cultures. Here, we set out to study the dynamics and stability of parasite genetic and phenotypic profiles as field isolate parasites were adapted in continuous cultures. Parasites collected from three different patients presenting with uncomplicated malaria were adapted and maintained in drug-free continuous cultures. Aliquots from the continuous cultures were collected every 24–48 hours for analyses. Each aliquot was treated as a separate parasite sample. For genetic analysis, microsatellite (MS) typing and single nucleotide polymorphism (SNP) analyses of 23 drug resistance markers were done. The 50% inhibitory concentrations (IC_50_) for some of the samples were also established for four antimalarial drugs. Samples from each patient (parasite-line) were compared as they were passed through the continuous culture. Data revealed genotypic and phenotypic profiles for the three parasite-lines fluctuated from one generation to the next with no specific pattern or periodicity. With few exceptions, multilocus analysis revealed samples from each parasite-line had high genetic diversity with unique haplotypes. Interestingly, changes in MS and SNP profiles occurred simultaneously. The difference in the IC_50_s of samples in each parasite-line reached statistical significance. However, phenotypic changes did not correspond or correlate to genotypic changes. Our study revealed parasite genetic and phenotypic characteristics fluctuates in short- and long-term cultures, which indicates parasite genetic information obtained even in short cultures is likely to be different from the natural infection parasites.

## Introduction

*Plasmodium falciparum in vitro* culture system has revolutionized malaria research, underpinning numerous aspects of malaria studies including drug susceptibility testing, parasite genetics, immunology, vaccine and drug discovery, and much more [1]. Establishment of reference parasite strains and clones with known genotypic and phenotypic characteristics further facilitated these studies [2–5]. Biological resource centers such as the Malaria Research and Reference Reagent Resource Center (MR4) supplies standard reference *P*. *falciparum* strains and clones to scientist all over the world. Once distributed, it is expected that the genotypic and phenotypic characteristics of each specific strain or clone will remain unchanged after culture adaptation.

*Plasmodium falciparum* reference strains and clones continue to undergo a series of short- and long-term continuous culture for distribution by the biological resource centers and other laboratories. On arrival at end-user laboratories, these parasites maybe culture adapted to build stable reservoirs for sustainable access. An account of *P*. *falciparum* culture systems across laboratories underscores variability in practice and reagents, such as variation in serum for media enrichment which is obtained from volunteers [6]. These conditions present inherent variability in culture systems. Most of the currently available reference strains and clones are over 20 years old, generating debate on how adequately they represent the present day field isolate parasites, which are widely genetically diverse [7]. This is disconcerting especially in the controlled human malaria infection trials where clonal parasite-lines such as NF54 and its clone 3D7 are used for testing candidate vaccines and antimalarial drugs.

Multilocus genotyping is used to investigate *P*. *falciparum* parasite genetics, population structure and dynamics in clinical or epidemiological field studies. Immunologically neutral polymorphic microsatellite loci such as those described by Anderson and colleagues [8], and surface antigen loci such as merozoite surface proteins 1 and 2 genes (*msp1* and *msp2*) [9, 10] are commonly used for multilocus genotypic typing. Using *msp1* and *msp2* to study parasite population dynamics in patients, Farnert and colleagues demonstrated that there were extensive dynamics of *P*. *falciparum* densities, life cycle stages and parasite genetic profiles in asymptomatic children that were followed for 5 days with frequent blood sampling [11]. This study was conducted in a high malaria transmission area in Tanzania. The analyses of the parasites revealed parasite densities and maturation stages fluctuated every 48 hours, with a gradual shift into more mature forms. Numerous genotype patterns were observed in each subject over the study period, and only few samples had identical profiles. Up to six alleles in *msp1* and *msp2* genes were found in samples collected six hours apart in the same individual. However, it is important to interpret this data (parasite genetic profiles) with caution since data obtained using antigenic loci such as *msp1* and *msp2* might reflect natural selection (due to host immunity) rather than population history [8, 12].

Multiply infected field isolates contain more than one parasite clones with distinct genotype and phenotype [13]. It is not clear whether *in vitro* cultures select for individual clones in multiply infected field isolates as they are culture adapted, and the eventual stability of genetic and phenotypic characteristics of parasite either as a polyclonal or monoclonal sample in continuous culture. In most laboratories, parasites are culture adapted for *in vitro* analysis and maybe kept in continuous cultures through multiple generations. However, the dynamics of the parasites in culture has not been well studied yet it might have significant impact on the genetic and phenotypic characteristic of the parasites. Here, we set out to study the dynamics of parasite genetic and phenotypic profiles as field isolate parasites were adapted in continuous culture over multiple generations. The stability of parasites genotypic and phenotypic characteristics in continuous culture were analyzed.

## Materials and Methods

### Ethical Statement

Ethical review boards of the Kenya Medical Research Institute (KEMRI) and Walter Reed Army Institute of Research (WRAIR) reviewed and approved this study under protocol numbers KEMRI SSC #1330 and WRAIR #1384. Patients presenting with uncomplicated malaria at the study site were enrolled into the study after providing informed consent per the study protocol. All potential study subjects provided written informed consent before screening, enrollment and had to pass an assessment of understanding. Protocol used in this study complied with International Conference on Harmonization Good Clinical Practice (ICH-GCP) guidelines. This study was conducted in accordance with the Declaration of Helsinki and the Belmont Report including all federal regulations regarding the protection of human participants as described in 32 CFR 219 (The Common Rule). Internal policies for human subject protections and the standards for the responsible conduct of research of the US Army Medical Research and Materiel Command were also followed. WRAIR holds a Federal Wide Assurance from the Office of Human Research Protections under the Department of Health and Human Services. Accordingly, all key study personnel in both studies were certified as having completed mandatory human research ethics education curricula and training under the direction of the WRAIR IRB Human Subjects Protection Program.

### Samples

Patient enrollment and sample collection have previously been described [14]. Red blood cells (RBCs, group “O”) for parasite culture were obtained from non-immune subjects who had no history of visiting malaria endemic regions within the country and had not donated blood for the last 6 months per the study protocol (WRAIR #1919). The RBCs were washed three times to eliminate white blood cells prior to use. Tissue culture media was prepared as previously described [15]. Briefly, RPMI 1640 basic media consisted of RPMI 1640 powder (10.4 grams, Gibco-Invitrogen), with 2 grams of glucose and 5.95 grams HEPES (H4034, Sigma) dissolved to homogeneity in 1 liter of de-ionized water and sterilized with a 0.2 μM filter. Tissue culture RPMI 1640 media, consisted of RPMI 1640 basic media with 10% (vol/vol) human ABO pooled plasma, 3.2% (vol/vol) sodium bicarbonate and 4 μg/mL hypoxanthine. The tissue culture media was stored at 4°C and used within 2 weeks.

### *Plasmodium falciparum in vitro* culture

Parasites were maintained in continuous culture as previously described [16]. Briefly, 2–3 mL isolate obtained from subjects were centrifuged at 8.0 *g* for 5 minutes and serum removed. This was followed by three cycles of washes in 7 mL of tissue culture media. Isolates at greater than 0.01% parasitemia were then adjusted to 0.01% at 6% hematocrit, gassed with a mixture of 90% nitrogen, 5% Oxygen and 5% carbon dioxide, and incubated at 37°C. Isolates at less than 0.01% parasitemia were not adjusted in cultured.

Reference clones from the liquid nitrogen library were revived for culture by first thawing at 37°C. The sample volume was noted and incubated in 5 volumes of 12% sodium chloride for 5 minutes in a 50 mL centrifuge tube. Secondly, 9 volumes of 1.6% sodium chloride were then added, gently mixed and centrifuged at 8.5 g for 3 minutes. The pellet was then re-suspended in 9 volumes of 0.9% sodium chloride and 0.2% dextrose and centrifuged at 8.5 g. The pellet was suspended in 4.5 mL tissue culture media, 0.2 mL of RBCs pellet added and incubated at 37°C after flushing with gas mixture (nitrogen 90%, Oxygen 5% and carbon dioxide 5%).

Media was changed at each cycle after 24 hours, parasitemia monitored after every 48 hours and aliquots taken for molecular analyses. After 1–2 weeks in culture, the isolates growing to greater than 3% parasitemia were analyzed for *in vitro* drug sensitivity by malaria SYBR Green I assay technique [15, 17]. A subset of the culture was split to 0.01% and parasites monitored to adapt to *in vitro* culture with the continuation of 48 hourly sample aliquoting; the remainders were cryopreserved. Each sample was monitored and aliquots at each cycle (24–48 hours) obtained for a period of between 60 and 90 days.

### *In vitro* drug testing

The division of experimental therapeutics of the WRAIR supplied the following drugs: chloroquine diphosphate (CQ), dihydroartemisinin (DHA), artemether (AT), mefloquine (MQ) and lumefantrine (LU). SYBR green I assay technique [18] with additional modification [19] was used for drug susceptibility testing of the parasites. Drugs were dissolves in dimethyl sulfoxide before performing a 2-fold serial dilution in tissue culture media to dose ranges of DHA (700 nM to 0.69 nM), LU (378 nM to 0.37 nM), MQ (1200 nM to 1.1 nM), AT (670 nM to 0.65 nM) and CQ (883 nM to 3.79 nM); 3.2 μL of the drug diluents were aliquoted into 384-well plates. Initial day zero field samples at >1% parasitemia, as well as reference clones and culture adapted field isolates attaining parasitemia of 3–8% were diluted to 0.6% parasitemia and 25 μL added to drug pre-dosed plates and incubated at 37°C. After 72 hours, lysis buffer containing (per liter) 100mMTris-HCl, 10mMEDTA, 0.016% Saponin, 1.6% triton X-100 and 20X SYBR Green I dye was added and the sample incubated for 24 hours in dark. The relative fluorescence units were read using the Tecan Genios Plus system with excitation and emission wavelengths of 485 nm and 535 nm respectively. The 50% inhibition concentration (IC_50_) values for each drug were calculated as previously described [17]. Each assay was performed in four replicates.

### *Plasmodium falciparum* DNA extraction

Parasite genomic DNA for the initial day zero field samples and the subsequent 48 hourly aliquots, were extracted from 200 μL of whole blood (for patient-derived samples) or (for culture-adapted parasites) using the QIAamp DNA Blood Mini Kit (Qiagen, Valencia, CA, USA) as per the manufacturer’s instructions. The DNA was stored at -20°C.

### Microsatellite genotyping

Parasite genomic DNA was used as template for amplification of twelve microsatellite (MS) loci distributed on seven of the 14 different chromosomes as previously described [8, 12]. The loci are Polyα (Chr4), TA42 (Chr5), TA81 (Chr5), TA1 (Chr6), TA109 (Chr6), TA87 (Chr 6), TA40 (Chr10), 2490 (Chr10), ARAII (Chr11), Pfg377 (Chr12), PfPk2 (Chr 12), and TA60 (Chr13). Hemi-nested PCR with fluorescent end-labeled primers were used for the MS amplification before electrophoresis with Genescan-500 LIZ labeled size standards on an ABI 3500xL capillary sequencer (Applied Biosystems, Carlsbad, CA, USA).

### Scoring of microsatellite allelic length variation and assessment of multiple infections

Multiple infections (MIs) were defined as those in which one or more of the twelve loci showed multiple alleles. GeneMapper v 4.0/4.1 (Applied Biosystems, Foster City, CA, USA) was used to automate measurement of allele length and to quantify peak heights. Multiple alleles per locus were scored if the peak height corresponding to minor alleles were ≥ 33% the height of the predominant allele in the isolate. Allele scoring was determined for each parasite clone where the largest among all the alleles was considered the multiplicity of infection of that sample.

### Estimation of genetic diversity in parasite populations

Genetic diversity was measured by the number of alleles per locus and expected heterozygosity based on allele frequency data in each parasite population. In the cases where multiple alleles were present at a locus, only the predominant allele, defined as the allele with the highest peak height in electropherograms was used for allele frequency calculation. This procedure is appropriate for estimation of population allele frequency if the composition of PCR products is representative of the composition of templates. The unbiased expected heterozygosity, *H*_*e*_ was calculated as:

$$H_{e}=\left[n/\left(n-1\right)\right]\left[1-\sum pi^{2}\right]$$

Where n is the number of isolates genotyped and pi is the frequency of the *i*^*th*^ allele. This was estimated using GenAlEx v2.2 and confirmed using GENEPOP software.

### SNP genotyping analysis

Genomic DNA extracted from the samples was assayed for 30 single nucleotide polymorphisms (SNPs) associated with drug resistance using PCR-based single base extension on Sequenom MassARRAY system (Sequenom, San Diego, CA, USA) as previously described [20]. These includes the *P*. *falciparum* multidrug resistance gene (*pfmdr1*; 86, 184, 1034, 1042, 1246), the *P*. *falciparum* chloroquine resistance gene (*pfcrt;* 72, 76, 356, 371), the *P*. *falciparum* dihydrofolate reductase gene (*pfdhfr*; 16, 59, 108, 164), the *P*. *falciparum* dihydropteroate synthase gene *(pfdhps*; 463, 437, 581, 613), the *P*. *falciparum* cytochrome b gene (*pfcytb*; 268, 284, 356), and the *P*. *falciparum* multidrug resistance protein 1 gene (*pfmrp1*; 437, 876, 1390) The primers used in the analysis of the 30 SNPs are shown in S1 Table.

### Network analysis

To determine the relatedness of the different parasite generations in each parasite line, the median-joining network was constructed in NETWORK version 4.6.1.1 (http://www.fluxus-engineering.com/sharenet.htm) to visualize the relationships among different alleles of the 12 MS markers. Loci that failed to amplify were assigned a null value (99) for analysis.

### Statistical analysis

To determine parasite genetic diversity and population structure, the 12 MS were used to calculate allele frequencies, heterozygosity values, molecular variance, haplotypes, principal coordinates and fixation indices using GenAlEx software. Predominant alleles were used in calculation of allele frequencies. For *H*_*e*_ analysis, samples from each parasite-line were analyzed separately. For this comparison, the mean *H*_*e*_ values were compared by using a Mann-Whitney U statistics implemented in the statistical package GraphPad Prism (San Diego, CA, USA). The *p* value of ≤ 0.05 was considered statistically significant. Continuous data in the form of IC_50_s were expressed as a mean with a standard deviation. The IC_50_ data for samples in each parasite-line for each drug tested were compared using the non-parametric tests (1- way ANOVA Kruskal-Wallis with Bonferroni's Multiple Comparison Test). IC_50_s data was correlated to the genetic markers and considered significant when *p* < 0.05.

## Results

### Length of the continuous culture

Three different field isolates—H63, H73 and H74—each considered as a parasite-line were adapted in continuous culture and maintained through multiple generations. Sample aliquots for analysis were collected every 24–48 hours. The H63 parasite-line was cultured continuously for 90 days, the H73 for 63 days and the H74 for 76 days. Aliquots collected from each parasite-line during the continuous culture were treated as separate parasite samples, including the day 0 samples (natural infection). For the H63 parasite-line, 26 samples (generations) were collected through day 90 whereas for the H73 and H74, 16 and 17 samples were collected through day 63 and 76 respectively. DNA was extracted from all the samples collected for genetic analysis whereas IC_50_s were generated for some of the samples.

### Microsatellite Analysis and stability of parasite generations

Allelic data for at least 10 of the 12 MS was obtained in all parasite samples. S2 Table shows the normalized MS data for the samples analyzed. Multiply infected parasite samples were determined by the number of alleles per locus. Parasites with more than one allele at any of the locus were considered polyclonal. The three natural infections (day 0) parasites were polyclonal (S2 Table). As the parasites were maintained in the continuous culture, the number and sizes of MS alleles at each locus kept shifting (appeared, disappeared or reappeared). The changes in genetic profiles continued as the parasites were maintained in the continuous culture without indication of stabilizing. For example, in the H63 parasite-line at the Polyα locus, two distinct alleles (153 and 156) were present on day 0 but on day 6, these alleles shifted (180 and 183). However, on day 18, these alleles shifted back to 153 and 156, and stayed the same through day 31 (S2 Table). There was yet another shift (192 and 195) on day 38, which stayed the same through day 44. These allele shifts continued through day 90, with seemingly no particular pattern in periodicity. These patterns of shifts were present in all the alleles in all the three parasite lines. Interestingly, the allele shifts mostly occurred simultaneously in multiple genetic loci, generating identical parasite haplotypes. For example, in H63, parasites from day 18 to day 31 had identical haplotype. A different haplotype appeared from day 38 through day 44. Since the MS loci span across the *Plasmodium falciparum* genome, this may indicate shift in parasite genome or the predominant clone(s) as the sample passes from one generation to the next in culture. The H73 and H74 parasite-lines were more polymorphic than H63, and these polymorphisms stayed consistent from one generation to the next.

### Parasite genetic diversity

Parasite genetic diversity was determined for samples generated from each parasite-line (considered as a population) using MS alleles. In the loci with multiple alleles, only the predominant allele based on pherograms peaks was used for analysis (S4 Table). From the number of distinct alleles observed at each locus, the unbiased expected heterozygosity (*H*_*e*_) estimated per locus is shown in Table 1 for each of the population. In the H63 parasite population, 2490 and Pfg377 were the least polymorphic loci with 2 alleles whereas TA109 was the most polymorphic with 7 alleles. In H73 parasite population, ARA2, PfPK2, TA1, TA42 and Pg377 were the least polymorphic with 2 alleles whereas TA109 and TA87 were the most polymorphic with 4 alleles. In H74 parasite population, ARA2, 2490, TA42 and TA1 were the least polymorphic with 2 alleles each whereas TA81 loci was the most polymorphic with 6 alleles. The mean number of alleles in all the loci in H63, H73 and H74 parasite-lines were 3.9, 2.7 and 3.6 respectively. The mean *H*_*e*_ was 0.55, 0.43 and 0.54 in H63, H73 and H74 respectively. The alleles and *H*_*e*_ values show there was genetic diversity in each of the three parasite populations generated as the parasites were propagated through the continuous cultures.

**Table 1:** Parasite genetic diversity for the three parasite lines. He is the unbiased expected heterozygosity, N is the number of samples analysed, Na is the number of alleles and Fst is the fixation index.

			H63			H73			H74		
			*n = 26*			*n = 16*			*n = 17*		
LOCUS	CHR	N	Na	*H*_*e*_	N	Na	*H*_*e*_	N	Na	*H*_*e*_	Fst
**Polyα**	4	25	6.0	0.70	16	3.0	0.52	14	4.0	0.71	0.24
**TA42**	5	26	4.0	0.64	16	2.0	0.12	16	2.0	0.48	0.32
**TA81**	5	26	3.0	0.15	15	3.0	0.66	17	6.0	0.68	0.22
**TA1**	6	22	3.0	0.53	11	2.0	0.42	9	2.0	0.37	0.32
**TA87**	6	25	5.0	0.74	16	4.0	0.69	15	5.0	0.46	0.23
**TA109**	6	25	7.0	0.78	10	4.0	0.76	17	5.0	0.66	0.15
**TA40**	10	23	3.0	0.55	16	3.0	0.33	15	4.0	0.58	0.30
**2490**	10	24	2.0	0.51	15	3.0	0.35	17	2.0	0.43	0.31
**ARA2**	11	26	6.0	0.78	16	2.0	0.32	17	2.0	0.43	0.28
**Pfpk2**	12	24	3.0	0.54	16	2.0	0.23	17	4.0	0.48	0.15
**Pfg377**	12	25	2.0	0.15	14	2.0	0.42	14	3.0	0.56	0.09
**TA60**	13	26	3.0	0.58	16	2.0	0.32	12	4.0	0.68	0.35
**MEAN**		25	3.9	0.55	15	2.8	0.43	15.0	3.6	0.54	0.25
**SE**		0.4	0.5	0.06	0.6	0.2	0.06	0.7	0.4	0.03	0.02

### Multilocus genetic analysis

**Fig 1:**
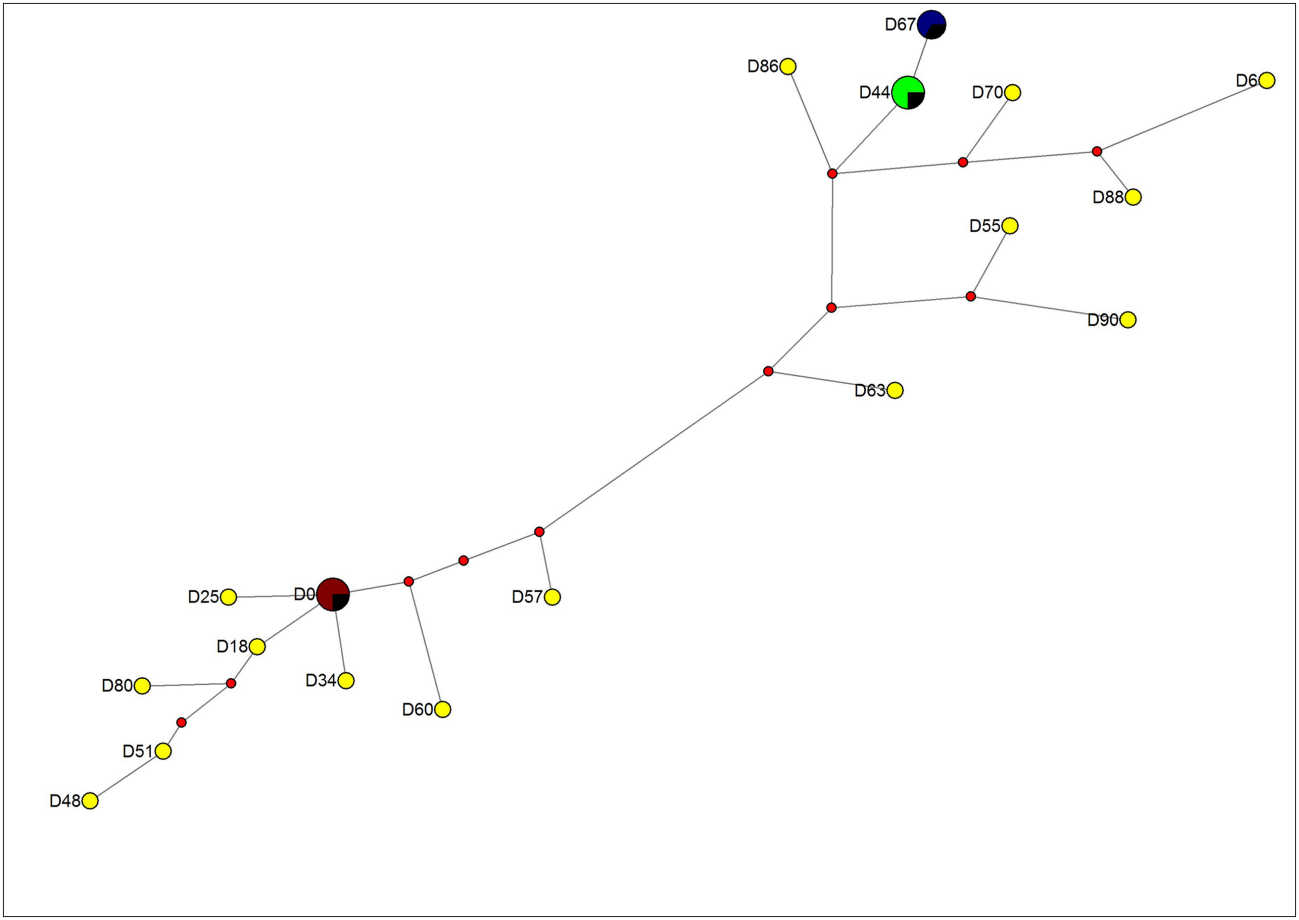
Median-joining network diagram for MS data. Diagram showing relationship of the different parasite generations in H63 parasite-line samples. The multilocus MS haplotypes profiles were constructed for each of the parasite generations using the 12 MS markers located across the *P*. *falciparum* genome. The 26 generations of the cultured *P*. *falciparum* field isolates analyzed formed 18 unique 12-loci microsatellite haplotypes. For allele sizes please refer to S4 Table. Each circle in the network represents a unique MS haplotype with the size of the circle being proportional to the number of isolates showing that particular haplotype. The red dots are hypothetical median vectors generated by the software to connect existing haplotypes within the network with maximum parsimony.

For multilocus genetic analysis, haplotypes were generated using the predominant alleles after normalization (S4 Table). Of the 26 samples in the H63 parasite-line, 18 unique haplotypes were formed using the 12 MS (Fig 1). Most samples had unique haplotype with the exception of 4-shared haplotypes. The first haplotype was shared between the H63 day 0 sample with samples from days 21, 31 and 34 while the second haplotype was shared between samples from days 38 and 44. The third haplotype shared between samples from days 40 and 84 was the same as the second haplotype except at only one locus. The fourth haplotype was shared between samples from days 74 and 78. The haplotype of the sample from day 67 was the same as the fourth haplotype except at one locus. The remainder of the samples had distinct haplotypes with varying degrees of similarities.

The 16 *P*. *falciparum* samples in the H73 parasite-line formed 15 unique 12-locus MS haplotypes. Similar to H63 parasite-line, the data revealed unique haplotypes from one generation to the next with the exception of one shared haplotype between day 0 and day 2. In the H74 parasite-line, the 17 samples analyzed formed 17 unique 12-loci MS haplotypes. Samples collected on days 18, 22, 47 and 55 had matching haplotypes except at one locus.

### Stability of drug resistance markers in continuous cultures

**Fig 2:**
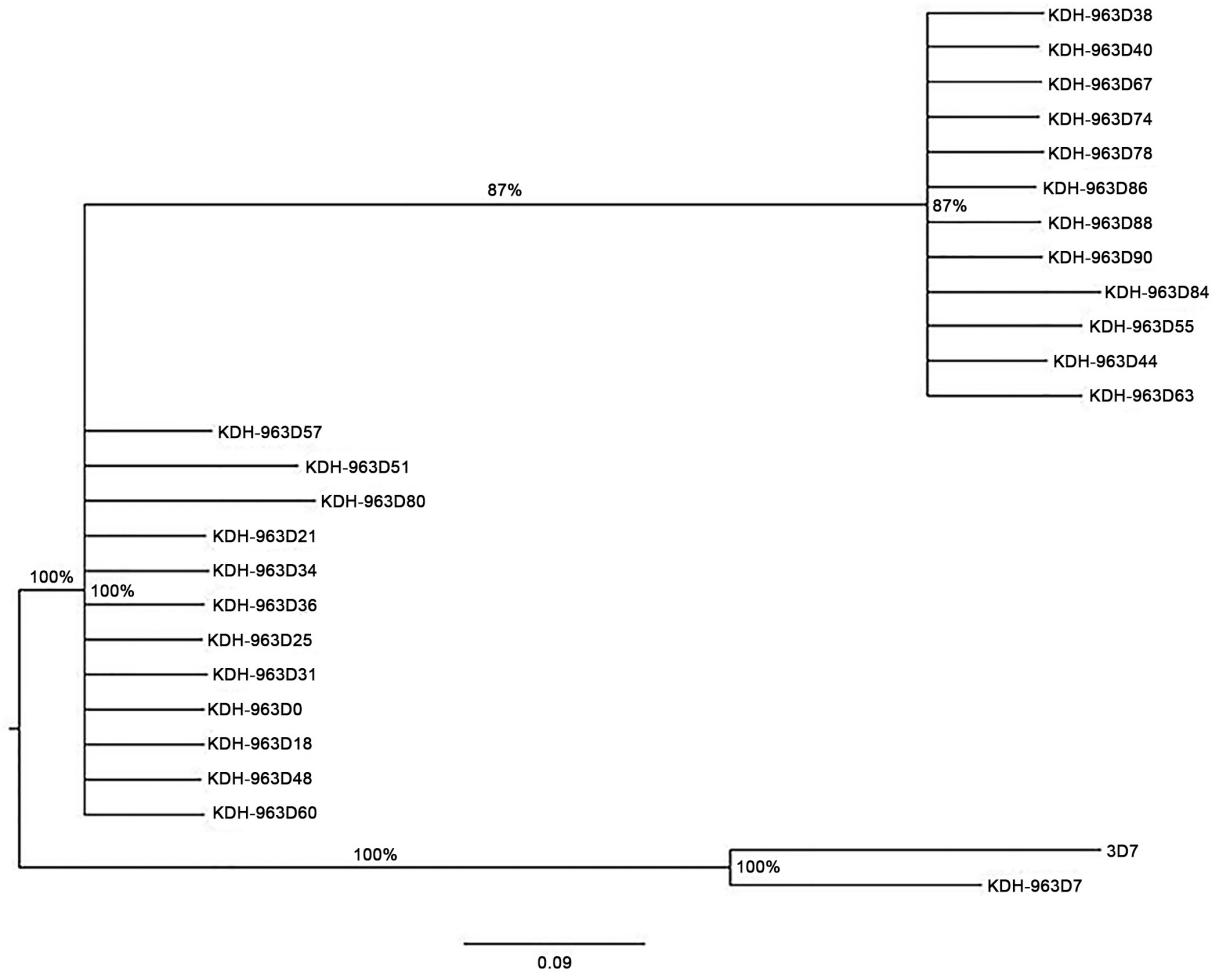
Phylogeny tree diagram for SNP data. Phylogeny tree constructed using SNP haplotypes of samples in H63 parasite-line. The SNP haplotype profiles are shown in S3 Table. Bayesian algorithm was used to infer the number of genetically related clusters from the individual SNP haplotype profiles generated using the 30-drug resistance SNPs.

Thirty SNPs associated with antimalarial drug resistance present in six different genes were analyzed in each of the parasite samples (S3 Table). Data revealed genetic haplotypes of the drug resistance markers shifted through the generations. Fig 2 shows a phylogenetic tree generated using haplotype SNP data from the H63 parasite-line. Interestingly, there was an overlap and similarities between points at which MS loci genetic shifts occurred and where the drug resistance SNP shifts occurred. For example, in the H63 parasite-line, parasites from day 18 to day 34 had the same MS genetic profiles and the drug resistance SNPs profile (Fig 2, S2 and S3 Tables). From day 38 to day 44, MS genetic profile shifted and likewise, the drug resistance SNPs profile shifted as well. Some parasite samples seemed to have mixed SNP genotype across several SNPs more than others. These mixed genotypes appeared to be transitional genotypes from one SNP to the next. For example, in the H63 parasite-line, day 57 sample, 7 out of 23 SNPs had mixed genotype whereas only 2 SNPs had mixed genotype on sample from the day before (day 55) and none on sample from the day after (day 60). All mixed SNPs carried SNP alleles found either in parasite samples collected before or after the sample with mixed genotype was collected. Similar genetic profiles and SNPs shifts were present in the H73 and the H74 parasite-lines.

### Parasite IC_50_s

Select parasite samples that attained parasitemia of 3–8% with greater than 70% ring stage parasites were used for *in vitro* susceptibility testing against the four antimalarial drugs as described in the methods section. The IC_50_ data was obtained for samples from the H63 and the H74 parasite-lines. The IC_50_s were obtained for 12 samples in the H63 parasite-line and 10 samples in the H74 parasite-line, including day 0 samples. On average, the IC_50_s data were collected every 8.5 and 8.7 days for the H63 and the H74 parasite samples respectively. Table 2 shows the mean IC_50_s obtained for each sample for the four drugs as well as the *p* value for each drug in a parasite-line. None of the drug showed any particular trend because as the parasites were kept in continuous culture, the IC_50_s fluctuated. The largest fluctuation seen from one generation to another was in the samples collected on day 85 to day 90 for chloroquine in the H63 parasite-line where the mean±SD IC_50_ increased from 2.8±0.8 to 49.8±1.8. However, none of the other drugs (artemether, lumefantrine, dihydroartemisinin) showed similar shift for the same set of samples. One-way analysis of variance revealed there was statistically significant difference (< 0.0001) in IC_50_s for the samples in the H63 and the H74 parasite-lines for all the drugs. Bonferroni’s multiple comparison tests further revealed that in the H63 parasite-line, chloroquine had the largest number of samples with significant differences in the IC_50_s when two samples were compared whereas dihydroartemisinin had the least (S5 Table). However, in the H74 parasite-line, Bonferroni’s multiple comparison test revealed dihydroartemisinin had the largest number of samples with significant difference in the IC_50_s when two samples were compared whereas chloroquine had the least (S6 Table).

**Table 2:** IC_50_s for select antimalarials. Values represent means and standard deviations for each assay run in four-replicates. For each parasite-line and each drug tested, p value is shown. ND = No Data.

	CQ	AT	LU	DHA
	mean±SD	mean±SD	mean±SD	mean±SD
**H 63 D 0**	21.5±1.6	2.19±0.9	6.94±2.4	3.41±1.2
**H 63 D 10**	8.6±2.5	3.42±1.9	4.48±0.6	1.87±0.2
**H 63 D 18**	9.0±0.3	3.29±0.7	8.52±1.5	2.59±0.7
**H 63 D 30**	16.1±2.3	2.61±1.3	10.92±0	1.72±0.3
**H 63 D 38**	16.0±0.0	6.80±0.2	6.44±0.4	5.34±0.2
**H 63 D 44**	48.5±0.0	9.06±0.7	5.10±0.4	1.43±0.9
**H 63 D 52**	55.7±2.3	4.77±1.6	4.93±1.7	4.25±1.2
**H 63 D 65**	39.9±0.0	8.01±3.2	5.43±0.2	1.41±0.0
**H 63 D 70**	5.5±1.1	3.80±1.9	3.76±2.9	3.82±2.2
**H 63 D 78**	15.2±0.2	9.18±1.0	31.21±0.1	3.08±0.5
**H 63 D 85**	2.8±0.8	2.99±1.0	29.57±0.0	1.67±0.1
**H 63 D 90**	49.8±1.8	10.77±0.2	19.70±0.1	1.10±0.1
	**P = 0.0152**	**P = 0.0005**	**P = 0.0092**	**P = 0.0002**
**H 74 D 0**	5.80±0.0	5.96±0.7	27.96±0.6	7.59±0.0
**H 74 D 12**	5.16±0.7	7.13±1.2	28.59±4.3	4.77±0.4
**H 74 D 19**	11.43±3.2	5.46±1.6	26.41±0.5	1.29±0.4
**H 74 D 28**	8.85±2.7	6.17±0.8	21.49±1.7	1.41±0.2
**H 74 D 35**	8.14±2.0	5.88±0.3	17.68±1.9	3.53±1.1
**H 74 D 44**	7.23±0.0	3.94±0.6	15.28±0.0	2.33±0.0
**H 74 D 52**	9.73±0.9	1.79±0.8	25.18±2.0	12.28±0.8
**H 74 D 60**	8.48±0.7	2.09±0.2	24.32±3.0	13.43±2.3
**H 74 D 68**	7.60±1.4	2.42±0.2	21.56±1.6	13.92±2.2
**H 74 D 76**	9.20±0.8	2.54±0.4	34.19±2.9	14.60±2.5
	**P = 0.0475**	**P = 0.0007**	**P = 0.0035**	**P = 0.0019**

### Correlation of the parasite IC_50_s with the MS profiles

One-way analysis of variance, Bonferroni’s multiple comparison tests was used to correlate parasite IC_50_s with parasite genotype. The MS profiles of parasite samples were analyzed and the changes in IC_50_s for all the four drugs compared. There was no correlation between the parasite IC_50_s with MS profile for the samples in the H63 and the H74 parasite-lines for all the four drugs tested. Some of the samples showed significant difference in IC_50_s yet they had similar MS profile whereas some samples had different MS profiles yet the IC_50_s were not significantly different. For example, samples H63 day 0 vs. day 18 or H63 day 38 vs. day 44 had significant difference in IC_50_s but had similar MS profiles. However, samples H63 day 0 vs. day 38 or H63 day 44 vs. day 90 did not have significantly different IC_50_s yet the MS profiles were different (S2 and S4 Tables).

### Association of parasite IC_50_s with drug resistance SNP profiles

Of the 30 drug resistance SNPs analyzed, in the H63 parasite-line, 13 SNPs changed once or more through the continuous cultures from one generation to the next whereas in the H74 parasite-line, only 7 SNPs changed (S3 Table). The data generated showed there was no association between the parasite IC_50_s and the drug resistance SNP profiles. For example, in the H63 parasite-line, samples from day 0 through day 36 had the same SNP genetic profile with exception of day 7. The SNP profile changed on day 38 through 44 at 7 SNP markers (MDR1246, MRPI 1390, MRPI 437, MRPI 437, MRPI 876, DHPS 436 and DHPS 613, S2 Table). However, Bonferroni’s multiple comparison test showed there was a significant difference in the chloroquine IC_50_s between sample H63 day 0 vs. day 10, and sample H63 day 0 vs. day 18 but no significance difference in the IC_50_s between sample H63 day 0 vs. day 30 and sample H63 day 0 vs. sample H63 day 38 (S3 Table). One of the largest the chloroquine IC_50_s change was from the sample H63 day 85 (IC_50_ = 2.8) and H63 day 90 (IC_50_ = 49.8) yet the MS profile in samples from day 84 through day 90 remained unchanged.

## Discussion

In this study, we have described *P*. *falciparum* parasite genetic and phenotypic characteristics in continuous *in vitro* culture. The data shows parasites’ genetic and phenotypic profiles fluctuate from one generation to another in continuous culture. Multilocus analysis revealed parasite samples from the same parasite-line showed genetic diversity and structure. SNPs associated with antimalarial drug resistance also fluctuated through generations. Interestingly, SNP and MS profiles changes occurred simultaneously in the same parasite generations, further confirming genomic shift of parasite clones through generations since the media used was drug free. The phenotypic characteristics of the parasites also fluctuated from one generation to the next in the continuous culture. However, the phenotypic changes did not coincide or correlate with genotypic changes.

Parasite infections in human host have complex dynamics where extensive changes in parasite genotypic profiles occur over time [21–24]. The appearance, disappearance and reappearance of strains and individual clones occur in natural infection over time [11, 21–24] and have been shown to occur in as little as 6 hour intervals [11]. The dynamics of *P*. *falciparum* infections in the human host are influenced by factors such as parasitemia, the number of clones, immunity, clinical status and transmission intensity [21–24]. Studying the dynamic of *P*. *falciparum* in culture should obviate the risk of human host factors influencing the data obtained. Indeed, *in vitro* drug susceptibility testing offers a more objectives approach in establishing parasite drug resistance profile because it is based on direct contact between parasites and the drug. In addition, several tests can be carried out with the same sample, and several drugs can be studied at the same time, including drugs that are still at the experimental stage [25]. However, the *in vitro* studies are complex and current methods have inherent methodological weaknesses in that different tests and methods that are not always comparable or reproducible are used. More so, even when the same test is used, the results are difficult to compare because of differences in laboratories. There is also variability in media reagents used across laboratories [25], which might also vary within the same laboratory. With some of these limitations in mind, we made every effort to ensure there was minimum variation of the media reagents used throughout the study. We also used controls in every experiment to ensure consistency in the data produced, and used modified SYBR Green I-based assay, which has improved sensitivity and reproducibility. The detailed analysis of the modified SYBR Green I-based assay has been reported elsewhere [Cheruiyot et al, manuscripts submitted for publication].

Even in the absence of drug pressure, our data shows parasite genotypic and phenotypic characteristics changed from one generation to another in the continuous culture. This shows similarity to natural infections in human host, where there is appearance, disappearance and reappearance of individual clones. Although factors such as parasitemia and immunity have been shown to influence the dynamics of parasite infection, *in vitro* data here suggests interplay within and between the parasites clones themselves play critical role in parasite dynamics. Although we made every effort to limit variability of the media reagents used, we cannot rule out the influence of culture media itself in influencing the dynamics of parasite genetic and phenotypic characteristics. It is also important to note since we used a PCR based assay, the limitations of the data presented are based on the sensitivity of the PCR assay and other inherent limitations such as detection of the predominant parasite clones only.

Genetic analysis of field isolates can be performed on parasite DNA obtained directly from a patient blood sample with malaria or after culture adaption. The first whole genome sequencing of human malaria *P*. *falciparum* was obtained from long-term culture adapted 3D7 clone [26]. Currently, *P*. *falciparum* whole genomes sequences are obtained from parasite clones or from field isolates [27]. In these studies, parasites are sequenced directly from the patient blood sample or after adapting to short- or long-term culture [25]. In this study, we have shown parasite genetic profile drastically fluctuates in short and long-term cultures. These changes seem to start immediately after the parasites are culture adapted. Furthermore, the genetic fluctuations continued through the entire period the parasites were in continuous culture. This data has critical implications for malaria studies including genetics, drug resistance and epidemiological analyses. Parasite genetic data obtained from samples that have been adapted in short- or long-term culture might not be the same as that found in the natural infection parasite, and might not be an accurate representation of the natural field parasite genetic in circulation. Even though the parasites in human host also fluctuate, the factors that might be the cause of fluctuations might be different compared to those *in vitro*. To further elucidate these observations, genetic analyses including whole genome sequencing of natural infection parasites and generations of short- and long-term cultures of the same parasite lineage will be critical in further quantifying the extent of genetic changes that occur as parasites are adapted in culture. It will be also important to culture adapt parasite beyond 90 days to determine if these genetic fluctuations stabilize overtime.

*In vitro* studies are technically complex and require highly skilled individuals. These studies are generally recommended to be conducted in reference laboratories in malaria-endemic countries in order to optimize the limited equipment and resources, and ensure standardization of methods [28]. Standardization is mostly done using reference strains and clones where historical World Health Organization (WHO) values for specific drugs against specific clones such as 3D7 and W2 are used as controls. It is evident from the data obtained in this study that parasites do not remain genetically and phenotypically stable in continuous culture. It will be important to investigate the stability of legacy clones such as 3D7 and W2 in continuous culture. Indeed, in our laboratory, we have experienced decline in the performance of cultures overtime. In some instances, we have seen drastic changes in the IC_50_s obtained or drastic changes in gametocyte production from a specific parasite stock (data not shown). When this happens, we are forced to either revive cultures from our master stock, request more from MR4 or request for an aliquot from one of our collaborating laboratories. Other researchers have expressed similar concerns and experiences (personal communications with Dr. Jason Regules of the WRAIR and Dr. Rhoel Dinglasan of Johns Hopkins Malaria Research Institute). These experiences might be explained by the genetic and phenotypic instability of continuous cultures, which might lead to critical phenotypic changes in parasite characteristics.

The mean IC_50_s for all the four drugs tested in the three parasite-lines changed significantly from generation to generation with no particular trend or periodicity. Some of the changes reached statistical significance but did not coincide or correlate to genetic changes. The presence of mixed population with different drug sensitivity has been considered as one of the drawbacks of the *in vitro* sample analysis [29]. In addition, it has been thought that the *in vitro* adaptation may stress parasite in ways that differ from population selection *in vivo*. If the changes in the IC_50_s coincided with genetic changes, then it would be reasonable to assume adaptation process selected for different clonal parasites with different genetic and phenotypic characteristics. However, the changes did not coincide. Our data (replicates) were highly reproducible with low standard deviations but the threshold of *in vitro* data has not been well defined. Therefore, although the difference in the IC_50_s from some parasite sample to another was significant as they were passed through generations, the implications of significance versus the IC_50_ threshold might not be obvious. Indeed, the historical WHO IC_50_ value for chloroquine resistance is 87 nM [30]. We have reported IC_50_ values of 13 nM for D6 (which is chloroquine sensitive) and 209.8 nM for W2 (which is chloroquine resistant) [14]. We have also reported IC_50_ values for lumefantrine of 8.0 nM for D6 and 45.1 nM for W2; lumefantrine susceptibility range has not been defined [30]. None of the data we are reporting here for chloroquine and other drugs shifted from a threshold that can be considered sensitive to a threshold that can be considered resistance based on analysis of reference strains, WHO standards and other reports [14, 29, 30]. Lack of standardized protocol, reliability and reproducibility has always been a concern for the *in vitro* assays [29]. Our data might explain the lack of reliability and reproducibility of *in vitro* analysis seen from laboratory to laboratory or even within the same laboratory.

## Conclusions

We have shown that in continuous cultures, parasites have complex dynamics where genetic and phenotypic characteristics remain unstable and has propensity to change throughout the culture life cycle. There is constant appearance, disappearance and reappearance of parasite clones in a culture. Although the change in the IC_50_ reached significance from one generation to another, the implications and magnitude might not be obvious. This study did not attempt to elucidate the role of culture media in these dynamics and it is likely that the sensitivity of method used for detection of genetic and phenotypic changes is critical as well. Further studies are required to elucidate our finding and to continue with discussions given the implications of our finding.

## Supporting Information

S1 Table Primers used for analysis of 30 SNPs in six different genes. The primers were generated by the design software, which is part of the Sequenom MassARRAY system. The 30 SNPs were designed into 3 pools. The primary PCR that is locus-specific PCR was then run using pools of 1^st^ PCRP and 2^nd^ PCRP to amplify the desired SNP loci. This secondary PCR uses mass-modified dideoxynucleotide terminator of an oligonucleotide primer (UEP_SEQ). The primer anneals immediately upstream of the polymorphic site of interest. The SNPs were added in a multiplexed single base pair extension (SBE) with dideoxynuleotides that are mass modified. The extended primers were then detected by Matrix- Assisted Laser Desorption/ Ionization, Time of Flight (MALDI-TOF) mass spectrometry in the Sequenom MassARRAY analyzer. 23 SNPs gave robust data and were used in subsequent analysis.
(DOCX)

S2 Table MS data for H63, H73 and H74. Raw data shown with all the alleles detected per locus.
(XLSX)

S3 Table SNPs data for H63, H73 and H74. SNP profiles shown for parasite samples analysed for each parasite-line. Both alleles in mixed infection shown. Samples with missing data are indicated as ND (No Data).
(XLSX)

S4 Table Predominant allele MS data for H63, H73 and H74. Predominant alleles were selected based on pherograms peaks; those with highest peaks were considered predominant.
(XLSX)

S5 Table Comparison of IC_50_s in H63 samples using Bonferroni's Multiple Comparison Test (1-way ANOVA Kruskal-Wallis). The difference of IC_50_s in parasite generations were compared for the 4 different drugs tested.
(XLSX)

S6 Table Comparison of IC_50_s in H74 samples using Bonferroni's Multiple Comparison Test (1-way ANOVA Kruskal-Wallis). The difference of IC_50_s in parasite generations were compared for the 4 different drugs tested.
(XLSX)
