# Supplementary material for: The Genotypic and Phenotypic Stability of Plasmodium falciparum Field Isolates in Continuous In Vitro Culture
Source: PLoS One. 2016 Jan 11;11(1):e0143565. doi: 10.1371/journal.pone.0143565 (PMC4713440; doi:10.1371/journal.pone.0143565)
Supplement: S1 Table — The primers were generated by the design software, which is part of the Sequenom MassARRAY system. The 30 SNPs were designed into 3 pools. The primary PCR that is locus-specific PCR was then run using pools of 1st PCRP and 2nd PCRP to amplify the desired SNP loci. This secondary PCR uses mass-modified dideoxynucleotide terminator of an oligonucleotide primer (UEP_SEQ). The primer anneals immediately upstream of the polymorphic site of interest. The SNPs were added in a multiplexed single base pair extension (SBE) with dideoxynuleotides that are mass modified. The extended primers were then detected by Matrix- Assisted Laser Desorption/ Ionization, Time of Flight (MALDI-TOF) mass spectrometry in the Sequenom MassARRAY analyzer. 23 SNPs gave robust data and were used in subsequent analysis. (DOCX) [file pone.0143565.s001.docx]

Table S1 Primers sequences used in SNPs analysis
